# Supplementary material for: Remanufacturing workshop management in blockchain and digital twin-based cloud remanufacturing service platform
Source: PLoS One. 2026 Mar 27;21(3):e0345757. doi: 10.1371/journal.pone.0345757 (PMC13028414; doi:10.1371/journal.pone.0345757)
Supplement: Attachment — Submitted filename: Response to the comments.docx [file pone.0345757.s002.docx]

Response to the comments

Dear Editor and Reviewer:

Thank you very much for your letter and the valuable comments, suggestions, and requirements concerning our manuscript. We highly appreciate the careful consideration of our work and the constructive guidance provided, which have been crucial for revising and improving the manuscript. We have thoroughly studied all the comments from the reviewer and the additional requirements from the journal, and have made corresponding revisions and improvements in strict accordance with all requirements.

Specifically, we have completely deleted Figures 6-9 and redrawn Figure 12, Figure 12 is an independently redrawn figure without any copyright issues, complying with the journal’s copyright requirements. Besides we have updated Data Availability statement, All files are available from the kaggle database DOI: https://doi.org/10.34740/kaggle/dsv/15036576.

**Reviewer #1:** We sincerely appreciate your positive evaluation of our manuscript (“The paper is well written and with novel information based on well searched reference sources”) and your constructive suggestions. We have carefully addressed each of your comments as follows:

1. There is no explicit found section named: Conclusions-Future work.

**[Response]:** Thank you so much for your suggestion. We have added an independent section entitled “Conclusions and Future Work” at the end of the manuscript. This section first systematically summarizes the core research findings, innovative points, and practical application value of this paper in the field of remanufacturing workshop management under the cloud remanufacturing service platform integrated with blockchain and digital twin technology. Subsequently, combined with the limitations of the current research, we clearly propose specific future research directions and expansion ideas, which improves the research closed loop of the paper, makes the presentation of research content more complete, and enhances the rigor of the research framework. This section has been marked in red in the revised manuscript.

2. The reference list could be improved

**[Response]:** Thank you so much for your suggestion. We have comprehensively optimized and supplemented the reference list. Specifically, we updated and added 5 latest high-quality literatures (published in 2024-2025) related to blockchain, digital twin, and cloud remanufacturing workshop management, strictly standardized the citation format of all references in line with PLOS ONE’s requirements, and verified the authority, timeliness, and completeness of each reference. These revisions further enhance the academic support and standardization of the paper. All adjustments to the reference list are marked in red in the revised manuscript.

3. Figure 12 could be made more readable.

**[Response]:** Thank you so much for your suggestion. According to the editor's opinion, we have completely deleted Figures 6-9. Now the original Figure 12 has been replaced by Figure 8. We have redrawn and comprehensively optimized Figure 8 to significantly improve its readability. Specifically, we increased the font size of the text and legends in the figure to ensure clarity (adjusted from 8pt to 10pt), adjusted the layout to make the information hierarchy more distinct, optimized the contrast of lines and color blocks to enhance visual effect, and added detailed annotations for key information to facilitate understanding. The optimized Figure 8 has been replaced at the corresponding position in the manuscript, and the relevant revisions are marked in red.
